# Supplementary material for: Altered microRNA Transcriptome in Cultured Human Liver Cells upon Infection with Ebola Virus
Source: Int J Mol Sci. 2021 Apr 6;22(7):3792. doi: 10.3390/ijms22073792 (PMC8038836; doi:10.3390/ijms22073792)
Supplement: Supplementary file 1 [file ijms-22-03792-s001.zip › Supplementary_File/D_ KEGG_Analysis_Results/16-30nt_pathway_Reston-96h-Huh7_vs_Control-96h-Huh7_up.mature_mirna_targets/hsa_pathwayResult.html]

| PathwayID | Definition | OriginalWebSite | Fisher-Pvalue | SelectionCounts | SelectionSize | Count | Size | FDR | Enrichment\_Score | GeneRatio | Genes |
| --- | --- | --- | --- | --- | --- | --- | --- | --- | --- | --- | --- |
| hsa04550 | Signaling pathways regulating pluripotency of stem cells - Homo sapiens (human) | http://www.genome.jp/kegg-bin/show\_pathway?hsa04550+648+100532731+1852+5595+6498 | 1.239684e-03 | 5 | 46 | 140 | 7906 | 4.140546e-01 | 2.906689 | 0.108696 | BMI1//COMMD3-BMI1//DUSP9//MAPK3//SKIL |
| hsa05135 | Yersinia infection - Homo sapiens (human) | http://www.genome.jp/kegg-bin/show\_pathway?hsa05135+5609+5595+4773+147179 | 5.038743e-03 | 4 | 46 | 120 | 7906 | 5.172933e-01 | 2.297678 | 0.086957 | MAP2K7//MAPK3//NFATC2//WIPF2 |
| hsa04380 | Osteoclast differentiation - Homo sapiens (human) | http://www.genome.jp/kegg-bin/show\_pathway?hsa04380+5609+5595+4773+4982 | 6.326599e-03 | 4 | 46 | 128 | 7906 | 5.172933e-01 | 2.198830 | 0.086957 | MAP2K7//MAPK3//NFATC2//TNFRSF11B |
| hsa04650 | Natural killer cell mediated cytotoxicity - Homo sapiens (human) | http://www.genome.jp/kegg-bin/show\_pathway?hsa04650+836+5595+4773+399694 | 6.861195e-03 | 4 | 46 | 131 | 7906 | 5.172933e-01 | 2.163600 | 0.086957 | CASP3//MAPK3//NFATC2//SHC4 |
| hsa05206 | MicroRNAs in cancer - Homo sapiens (human) | http://www.genome.jp/kegg-bin/show\_pathway?hsa05206+648+836+100532731+27165+5595+399694 | 8.644649e-03 | 6 | 46 | 310 | 7906 | 5.172933e-01 | 2.063253 | 0.130435 | BMI1//CASP3//COMMD3-BMI1//GLS2//MAPK3//SHC4 |
| hsa05214 | Glioma - Homo sapiens (human) | http://www.genome.jp/kegg-bin/show\_pathway?hsa05214+57118+5595+399694 | 9.292695e-03 | 3 | 46 | 75 | 7906 | 5.172933e-01 | 2.031858 | 0.065217 | CAMK1D//MAPK3//SHC4 |
| hsa04012 | ErbB signaling pathway - Homo sapiens (human) | http://www.genome.jp/kegg-bin/show\_pathway?hsa04012+5609+5595+399694 | 1.305560e-02 | 3 | 46 | 85 | 7906 | 5.932916e-01 | 1.884203 | 0.065217 | MAP2K7//MAPK3//SHC4 |
| hsa05161 | Hepatitis B - Homo sapiens (human) | http://www.genome.jp/kegg-bin/show\_pathway?hsa05161+836+5609+5595+4773 | 1.421058e-02 | 4 | 46 | 162 | 7906 | 5.932916e-01 | 1.847388 | 0.086957 | CASP3//MAP2K7//MAPK3//NFATC2 |
| hsa04660 | T cell receptor signaling pathway - Homo sapiens (human) | http://www.genome.jp/kegg-bin/show\_pathway?hsa04660+5609+5595+4773 | 2.174509e-02 | 3 | 46 | 103 | 7906 | 6.861899e-01 | 1.662639 | 0.065217 | MAP2K7//MAPK3//NFATC2 |
| hsa05167 | Kaposi sarcoma-associated herpesvirus infection - Homo sapiens (human) | http://www.genome.jp/kegg-bin/show\_pathway?hsa05167+836+5609+5595+4773 | 2.243888e-02 | 4 | 46 | 186 | 7906 | 6.861899e-01 | 1.648999 | 0.086957 | CASP3//MAP2K7//MAPK3//NFATC2 |
| hsa04659 | Th17 cell differentiation - Homo sapiens (human) | http://www.genome.jp/kegg-bin/show\_pathway?hsa04659+5595+4773+6097 | 2.401683e-02 | 3 | 46 | 107 | 7906 | 6.861899e-01 | 1.619484 | 0.065217 | MAPK3//NFATC2//RORC |
| hsa04668 | TNF signaling pathway - Homo sapiens (human) | http://www.genome.jp/kegg-bin/show\_pathway?hsa04668+836+5609+5595 | 2.703161e-02 | 3 | 46 | 112 | 7906 | 6.861899e-01 | 1.568128 | 0.065217 | CASP3//MAP2K7//MAPK3 |
| hsa04724 | Glutamatergic synapse - Homo sapiens (human) | http://www.genome.jp/kegg-bin/show\_pathway?hsa04724+27165+2901+5595 | 2.829195e-02 | 3 | 46 | 114 | 7906 | 6.861899e-01 | 1.548337 | 0.065217 | GLS2//GRIK5//MAPK3 |
| hsa04722 | Neurotrophin signaling pathway - Homo sapiens (human) | http://www.genome.jp/kegg-bin/show\_pathway?hsa04722+5609+5595+399694 | 3.157848e-02 | 3 | 46 | 119 | 7906 | 7.031474e-01 | 1.500609 | 0.065217 | MAP2K7//MAPK3//SHC4 |
| hsa05170 | Human immunodeficiency virus 1 infection - Homo sapiens (human) | http://www.genome.jp/kegg-bin/show\_pathway?hsa05170+836+5609+5595+4773 | 3.410577e-02 | 4 | 46 | 212 | 7906 | 7.119580e-01 | 1.467172 | 0.086957 | CASP3//MAP2K7//MAPK3//NFATC2 |
| hsa04926 | Relaxin signaling pathway - Homo sapiens (human) | http://www.genome.jp/kegg-bin/show\_pathway?hsa04926+5609+5595+399694 | 3.872917e-02 | 3 | 46 | 129 | 7906 | 7.609143e-01 | 1.411962 | 0.065217 | MAP2K7//MAPK3//SHC4 |
| hsa04370 | VEGF signaling pathway - Homo sapiens (human) | http://www.genome.jp/kegg-bin/show\_pathway?hsa04370+5595+4773 | 4.595193e-02 | 2 | 46 | 59 | 7906 | 8.526636e-01 | 1.337696 | 0.043478 | MAPK3//NFATC2 |
